# Mitochondrial Dysfunction and Immune Cell Infiltration in Diabetic Kidney Disease: A Mendelian Randomization and Multiomics Study

**DOI:** 10.1155/mi/5592084

**Published:** 2025-12-25

**Authors:** Tianyue Zhang, Junxia Wu, Jiazhi Zhang, Yepeng Hu, Yiming Zhao, Guangyun Mao, Jingjing Jiao, Jun Wang, Riqiu Chen, Chao Zheng

**Affiliations:** ^1^ Department of Endocrinology, The Second Affiliated Hospital, Zhejiang University School of Medicine, Hangzhou, Zhejiang, China, zju.edu.cn; ^2^ Department of Nutrition, Harvard T.H. Chan School of Public Health, Boston, Massachusetts, USA, harvard.edu; ^3^ Department of Nephrology, The Second Affiliated Hospital, Zhejiang University School of Medicine, Hangzhou, Zhejiang, China, zju.edu.cn; ^4^ Division of Epidemiology and Health Statistics, Department of Preventive Medicine, School of Public Health and Management, Wenzhou Medical University, Wenzhou, Zhejiang, China, wmu.edu.cn; ^5^ Department of Nutrition, School of Public Health, Zhejiang University School of Medicine, Hangzhou, Zhejiang, China, zju.edu.cn; ^6^ Department of Gastroenterology Surgery, The Second Affiliated Hospital, Zhejiang University School of Medicine, Hangzhou, Zhejiang, China, zju.edu.cn; ^7^ Department of Endocrinology, Lishui People’s Hospital, Lishui, Zhejiang, China

**Keywords:** diabetic kidney disease, immune cells, Mendelian randomization, mitochondrial dysfunction, single-cell RNA sequencing

## Abstract

**Background:**

Diabetic kidney disease (DKD) is a multifactorial complication of diabetes involving mitochondrial dysfunction and immune cell infiltration. However, the causal relationships remain unclear.

**Methods:**

We applied Mendelian randomization (MR) and single‐cell RNA sequencing (scRNA‐seq) to investigate the roles of mitochondrial gene expression and immune cells in DKD. Additionally, peripheral blood mononuclear cells (PBMCs) from DKD patients were analyzed for differential gene expression.

**Results:**

Higher expression of mitochondrial genes PCCB, ACADM, ADHFE1, OCIAD1, and FIS1 increased DKD risk, while genes like NT5DC2, ATP5MC3, and GLYCTK decreased risk. Immune traits, including human leukocyte antigen (HLA)‐DR + plasmacytoid dendritic cells (pDCs), mediated the effects of mitochondrial dysfunction on DKD. scRNA‐seq revealed significant downregulation of ATP5MC3, GLYCTK, and NT5DC2 in podocytes (PODOs) and tubular cells in DKD kidneys, alongside increased infiltration of helper T cells, B cells, dendritic cells (DCs), and plasma cells. PBMC analysis highlighted the upregulation of proinflammatory genes (CXCL2, CXCL3, and others) in DKD patients.

**Conclusion:**

This study highlights the complex interplay between mitochondrial dysfunction and immune cell infiltration in DKD pathogenesis. Key mitochondrial genes and immune traits identified here offer novel therapeutic targets such as ATP5MC3, GLYCTK, and DC pathways.

**Summary**

1. Identified 14 mitochondrial‐related genes and three immune cell traits associated with diabetic kidney disease (DKD) risk.
2. Demonstrated mitochondrial genes like PCCB, ACADM, and FIS1 increase DKD risk, while ATP5MC3, GLYCTK, and NT5DC2 reduce risk.
3. Showed immune traits such as human leukocyte antigen (HLA‐DR) + plasmacytoid dendritic cells (pDCs) mediate the impact of mitochondrial dysfunction on DKD.
4. Single‐cell RNA sequencing (scRNA‐seq) revealed specific mitochondrial gene downregulation and increased immune cell infiltration in DKD kidneys.
5. Findings provide novel insights into the interplay of mitochondrial dysfunction, immune response, and DKD progression, highlighting potential therapeutic targets.

## 1. Introduction

Diabetic kidney disease (DKD) affects ~40% of individuals with diabetes and is a leading cause of chronic kidney disease (CKD) worldwide. Although DKD progression can culminate in end‐stage renal disease (ESRD), most patients succumb to cardiovascular complications or infections before requiring kidney replacement therapy [1, 2]. The disease progresses through stages of glomerular hyperfiltration, albuminuria, declining glomerular filtration rate (GFR), and ESRD, but current treatments fail to halt its progression, necessitating novel therapeutic strategies [3].

Mitochondrial dysfunction is central to DKD pathogenesis. It is characterized by increased reactive oxygen species (ROS) generation, impaired biogenesis, altered bioenergetics, and disrupted mitochondrial dynamics [4, 5]. These abnormalities have been implicated in podocyte (PODO) injury and glomerular dysfunction through mechanisms such as excessive mitochondrial fission and oxidative stress [6, 7]. Despite advancements in understanding mitochondrial roles in DKD, the precise causal relationships remain underexplored.

In parallel, renal inflammation and immune cell infiltration are increasingly recognized as key contributors to DKD [8]. Mitochondrial dysfunction may activate inflammatory pathways by disrupting energy metabolism, promoting ROS generation, and triggering immunosenescence [9, 10]. However, the interplay between mitochondrial dysfunction, immune cell activation, and DKD pathogenesis is not fully understood.

To address these gaps, this study employs Mendelian randomization (MR) to infer causal effects and single‐cell RNA sequencing (scRNA‐seq) to resolve cell‐specific gene expression profiles. MR uses genetic variants as instrumental variables, reducing confounding and providing robust causal inferences [11]. scRNA‐seq enables the identification of rare cell populations and cell‐specific functions, offering high‐resolution insights into kidney pathology [12]. This study investigates the hypothesis that mitochondrial dysfunction contributes to DKD by driving immune cell infiltration and activation. By integrating MR, scRNA‐seq, and peripheral blood mononuclear cell (PBMC) analysis, we aim to uncover novel therapeutic targets for this complex disease.

## 2. Methods

### 2.1. Study Design

The study was structured into five key phases (Figure 1A): Phase 1 involved identifying instrumental variables. Phase 2 aimed to identify causal links between mitochondrial‐related genes and DKD, with these associations subsequently verified in an independent cohort to ensure robustness. MR required meeting three core assumptions: relevance, independence, and exclusion restriction. Phase 3 examined the role of specific immune cell types as intermediaries in the pathway from mitochondrial dysfunction to DKD. Phase 4 utilized a single‐cell dataset from the Gene Expression Omnibus (GEO) database to validate findings of mitochondrial dysfunction and immune cell abnormalities in DKD at a single‐cell resolution. Finally, Phase 5 employed PBMC transcriptional profiling analysis to investigate whether PBMCs from DKD patients secrete abnormal inflammatory factors. This study elucidated the hypothesis that renal mitochondrial dysfunction leads to immune cell infiltration, ultimately causing DKD (Figure 1B).

Figure 1Study design and proposed mechanism linking mitochondrial dysfunction to immune activation in DKD. (A) Overview of the study design. (B) Proposed mechanism based on multiomics findings. DKD, diabetic kidney disease; eQTL, expression quantitative trait locus; FinnGen, Finnish Genome Center; GTEx, Genotype‐Tissue Expression Project.

**Figure:**
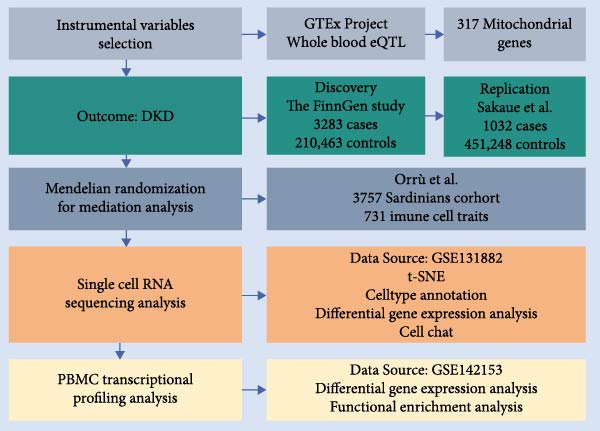
(A)

**Figure:**
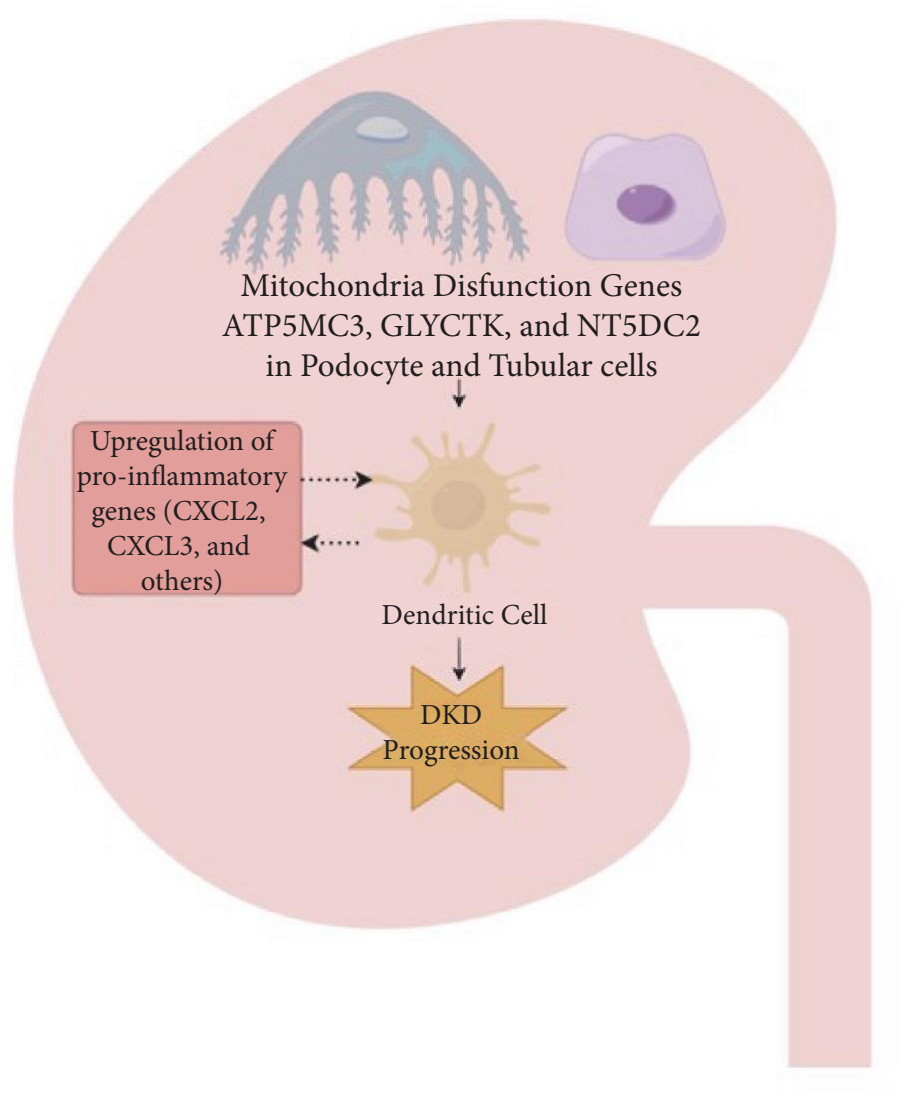
(B)

### 2.2. Data Sources

Blood expression quantitative trait loci (QTL) data were obtained from the Genotype‐Tissue Expression (GTEx) Portal (https://www.gtexportal.org/home/). Mitochondrial‐related genes were identified from MitoCarta3.0, which includes an updated inventory of 1136 human mitochondrial genes (https://www.broadinstitute.org/mitocarta/mitocarta30-inventory-mammalian-mitochondrial-proteins-and-pathways). Genetic variants reaching a significance (*p*‐value < 1e^−5^) were identified as potential genetic instruments. Variants in strong linkage disequilibrium (LD) were excluded by applying a criterion of *r* ^2^ < 0.1 within a 100 kb window using PLINK software. Consistent with previous studies, we applied a threshold of *p*  < 1 × 10^−5^ [13, 14] together with an LD clumping threshold of *R* ^2^ < 0.1 [15] to identify SNPs as suggestive instruments, ensuring an adequate number of variants for analysis. F‐statistics were computed for each variant to evaluate instrument strength, with a threshold of *F* > 10 to mitigate bias. Ultimately, 317 mitochondrial‐related genes met the above criteria.

DKD outcome data were sourced from the FinnGen study (finngen_R9_DM_NEPHROPATHY_EXMORE data; https://www.finngen.fi/en/access_results). To validate MR findings, an independent DKD dataset (ebi‐a‐GCST90018832) was employed (https://gwas.mrcieu.ac.uk/). GWAS data of 731 immune traits, including subsets of T cells, B cells, macrophages, and dendritic cells (DCs), were derived from a cohort of 3757 Sardinians (https://www.ebi.ac.uk/gwas/publications/32929287). The single‐cell analysis utilized a publicly available scRNA‐seq dataset (GSE131882) from the GEO database. Kidney cortex samples were taken from three nondiabetic controls and three individuals with diabetes following nephrectomy for renal mass. Diabetic patients exhibited elevated A1c, mesangial sclerosis, and glomerular basement membrane thickening. Estimated GFR ranged from 56 to 85 mL/min/1.73 m^2^. PBMC transcriptional profiling data were obtained from a publicly available dataset (GSE142153) comparing healthy controls with DKD patients.

### 2.3. Mediation MR Analysis

A two‐step mediation MR analysis was conducted to explore whether immune cells mediate the causal pathway from mitochondrial dysfunction to DKD. This process included calculating two MR estimates: the causal effect of mitochondrial dysfunction on immune cells (*β*1) and the causal effect of immune cells on DKD (*β*2). These estimates were multiplied to determine the indirect effect. The total effect of mitochondrial dysfunction on DKD risk was also calculated to contextualize the mediation analysis.

### 2.4. Sensitivity Analysis

Several sensitivity analyses were employed to ensure robustness and validity of findings. MR‐Egger regression was used to detect and correct for potential pleiotropic effects. Leave‐one‐out analysis assessed the influence of individual genetic variants on overall results. Cochran’s Q test evaluated heterogeneity among genetic instruments, ensuring that observed associations were not driven by outlier variants.

### 2.5. Single‐Cell Analysis

Raw scRNA‐seq data from the GEO database were processed using standard bioinformatics pipelines. Quality control steps included filtering low‐quality cells, normalization, and batch effect correction. Distinct cell populations within kidney tissues were identified and annotated based on established markers and clustering algorithms. Differential gene expression analysis highlighted mitochondrial‐related differentially expressed genes (DEG) (fold change > 2, *p*  < 0.05) between DKD and healthy samples within specific cell types. This analysis provided insights into mitochondrial dysfunction at a cellular level.

### 2.6. Statistical Analysis

All analyses were conducted using TwoSampleMR (Version 0.5.8) and MendelianRandomization (Version 0.8.0) in R Software 4.3.2 (https://www.R-project.org). The Wald ratio estimate was calculated for each genetic variant and summarized using the inverse‐variance weighted (IVW) method. Random‐effects IVW models were applied in cases of heterogeneity; otherwise, fixed‐effect models were used. Benjamini–Hochberg correction was applied for multiple testing, with significance thresholds set at *p*  < 0.05 and FDR < 0.1. Visualization was performed using ggplot2 (Version 3.4.4) in R.

## 3. Results

### 3.1. Causal Effects of Mitochondrial Gene Expression on DKD

**Figure 2:**
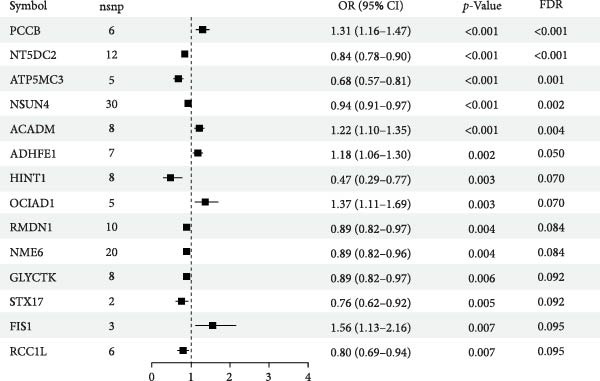
Causal effects of mitochondrial gene expression on DKD. CI, 95% confidence interval; OR, odds ratio.

Results for the causal effects of mitochondrial gene expression on DKD are shown in Figure 2. In total, 14 associations were identified at a nominal significance level (*p*  < 0.05, FDR < 0.1). After multiple testing correction, genetically predicted higher expression of PCCB, ACADM, ADHFE1, OCIAD1, and FIS1 was positively associated with DKD risk. Conversely, higher expression of NT5DC2, ATP5MC3, NSUN4, HINT1, RMDN1, NME6, GLYCTK, STX17, and RCC1L was inversely associated with DKD risk. Sensitivity analyses detected no evidence of horizontal pleiotropy or heterogeneity. Associations for PCCB, NT5DC2, ADHFE1, RMDN1, ATP5MC3, HINT1, GLYCTK, and RCC1L were replicated in an independent cohort (Supporting Information 1: Figure S1).

### 3.2. Causal Effects of Immune Cells on DKD

Results for the causal effects of immune cells on DKD are shown in Figure 3A. Three associations were identified at the nominal significance level (*p*  < 0.05, FDR < 0.1). Human leukocyte antigen (HLA)‐DR + plasmacytoid DCs (pDCs), DCs, and HLA‐DR on CD33‐ HLA‐DR + were positively associated with DKD risk. Heterogeneity was observed in these immune cells (*p*  < 0.001); however, no evidence of horizontal pleiotropy was detected. Associations for HLA‐DR on pDC and HLA‐DR on CD33‐ HLA‐DR + were replicated (Supporting Information 2: Figure S2).

Figure 3Mediation MR analysis. (A) Causal effect of immune cell traits on DKD. (B) Mediation effect of immune cell traits in causal effect of mitochondrial gene expression. Beta_All: The beta value of mitochondrial gene expression on DKD. Beta1: The beta value of mitochondrial gene expression on immune cell traits. Beta2: The beta value of immune cell traits on DKD. LCI, low 95% confidence interval; proportion: mediation effect; UCI, upper 95% confidence interval.

**Figure:**
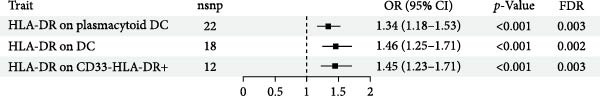
(A)

**Figure:**
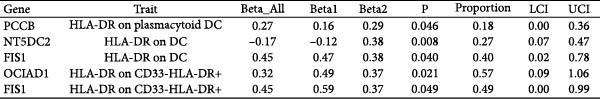
(B)

### 3.3. Mediation MR Analysis

A two‐step mediation MR analysis revealed that immune cells partially mediate the causal pathway from mitochondrial gene expression to DKD risk (Figure 3B). The causal effects of PCCB, NT5DC2, FIS1, and OCIAD1 on DKD were mediated by immune cell traits, with mediation proportions ranging from 18% to 57% (all *p*  < 0.05).

### 3.4. Changes in Mitochondrial Gene Expression in the Renal Cortex of DKD Patients

Ten kidney cell types (proximal convoluted tubule [PCT], complement factor H [CFH], loop of Henle [LOH], distal convoluted tubule [DCT], connecting tubule [CT], principal cell [PC], PODO, endothelium [ENDO], mesangial cell [MES], and leukocyte [LEUK]) were identified through unsupervised clustering and lineage‐specific markers (Figure 4A; Supporting Information 3: Figure S3A,B). DEGs and mitochondrial‐related DEGs in each cell type are presented in Supporting Information 4: Figure S4B and Figure 4B. Among mitochondrial genes with causal effects on DKD, ATP5MC3 and NT5DC2 were downregulated in PODOs, while GLYCTK was downregulated in LOH and PCT (Figure 4C–F; Supporting Information 4: Figure S4C,D), aligning with their inverse associations with DKD risk in MR analysis.

Figure 4Mitochondrial‐related DEGs in renal cortex of DKD patients. (A) Identification of kidney cell types. (B) Top three mitochondrial‐related DEGs in each cell type. (C) Heatmap of log2 fold change values for ATP5MC3, NT5DC2, and GLYCTK. (D‐F) Violin plot of ATP5MC3, NT5DC2, and GLYCTK (due to the small number of LEUK in the control group, differential analysis of LEUK was not performed). CFH, complement factor H; CT, connecting tubule; DCT, distal convoluted tubule; ENDO, endothelium; LEUK, leukocyte; LOH, loop of Henle; PC, principal cell; PODO, podocyte; MES, mesangial cell; PCT, proximal convoluted tubule.

**Figure:**
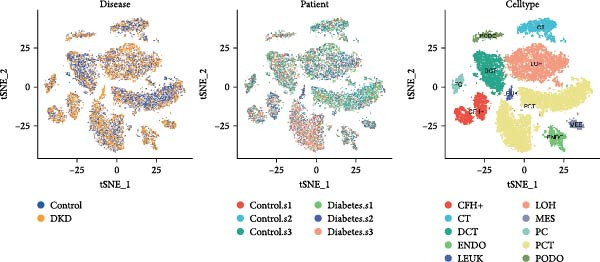
(A)

**Figure:**
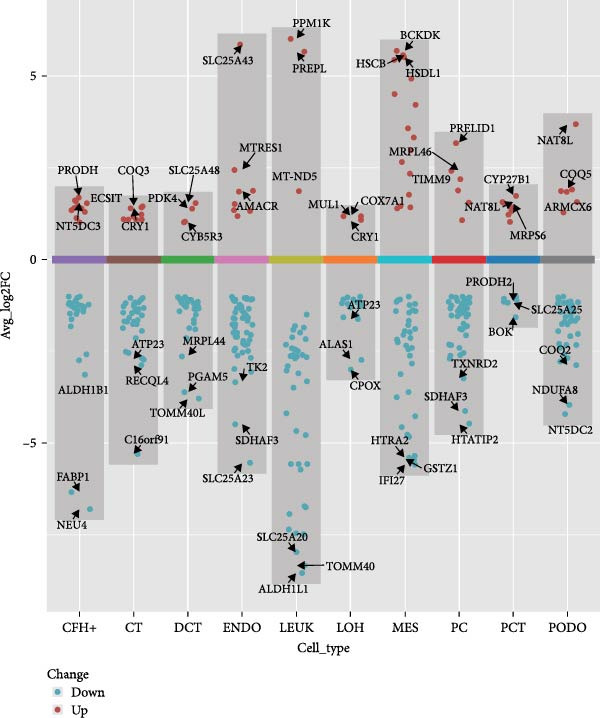
(B)

**Figure:**
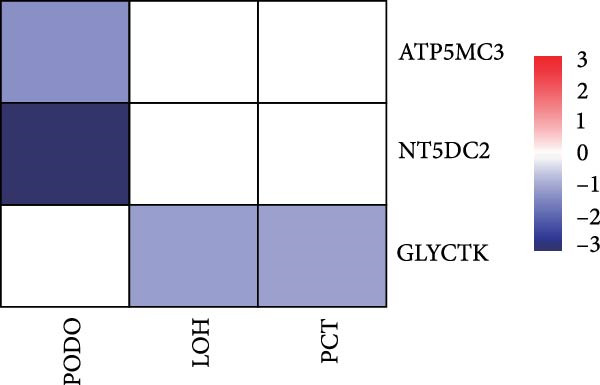
(C)

**Figure:**
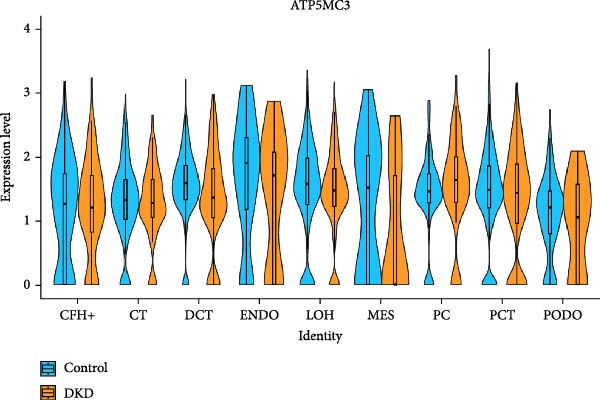
(D)

**Figure:**
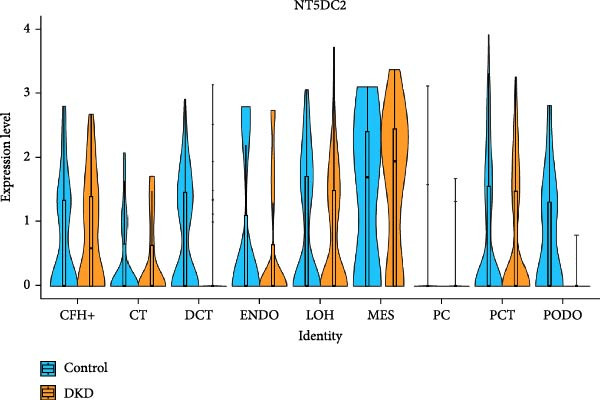
(E)

**Figure:**
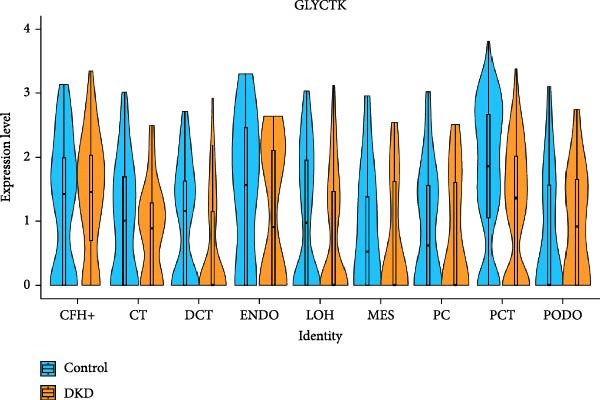
(F)

### 3.5. Changes in Immune Cell Infiltration in the Renal Cortex and Transcriptomic Landscape of PBMCs in DKD Patients

Although lLEUK‐parenchymal ligand–receptor signaling appeared weak in inference (Supporting Information 4: Figure S4E,F), LEUK cell counts in the renal cortex of DKD patients were elevated (Supporting Information 4: Figure S4A), with subclustering revealing increased counts of helper T cells, B cells, DCs, and plasma cells in diabetics (Figure 5A,B), suggesting that immune contributions may operate mainly via recruitment and activation rather than through stable local ligand–receptor interactions. PBMC analysis identified upregulation of proinflammatory genes (CXCL2, CXCL3, DEFA3, EMP1, HBD, SMAD7, SPRR1B) and downregulation of genes (CD246, KLHL34, MCF2L‐AS1, NOG, NRCAM, RAD54B, SLC4A10, VSIG1) in DKD patients (Figure 5C,D; Supporting Information 4: Figure S4G,H). These findings highlight the proinflammatory milieu in DKD and suggest potential therapeutic targets for intervention.

Figure 5Changes in immune cell infiltration in renal cortex of DKD patients and DEGs in PBMCs from DKD patients. (A) Identification of immune cell types in renal cortex of DKD patients. (B) Cell counts of immune cell types in renal cortex of DKD patients. (C) Heatmap of DEGs in PBMCs from DKD patients. (D) Violin plot of DEGs in PBMCs from DKD patients. PBMC, peripheral blood mononuclear cell.

**Figure:**
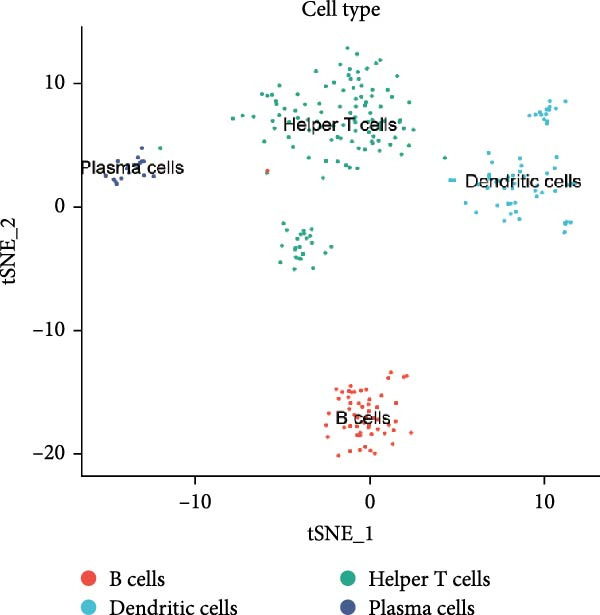
(A)

**Figure:**
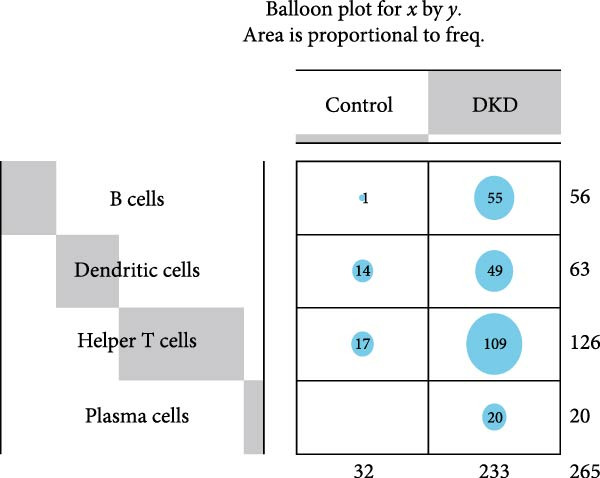
(B)

**Figure:**
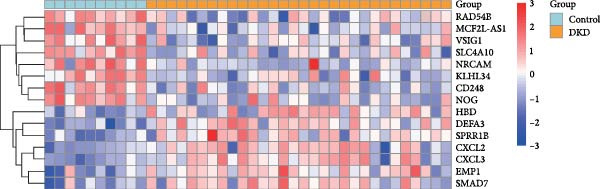
(C)

**Figure:**
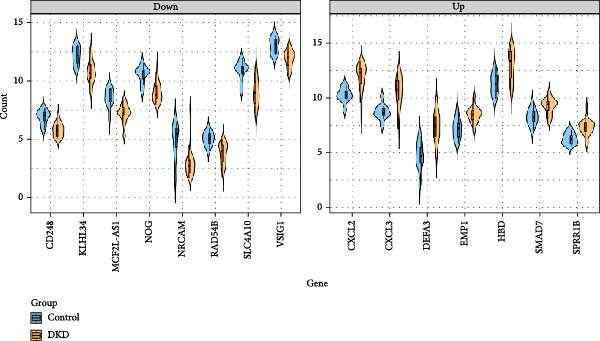
(D)

## 4. Discussion

This study provides novel insights into the complex interplay between mitochondrial dysfunction, immune cell infiltration, and the progression of DKD. By leveraging integrative MR, we identified key mitochondrial genes and immune cell traits implicated in DKD pathogenesis, offering potential therapeutic targets for early intervention.

Our findings emphasize the critical role of mitochondrial dysfunction in DKD. Several mitochondrial genes, including PCCB, ACADM, ADHFE1, OCIAD1, and FIS1, were associated with an increased risk of DKD, suggesting that elevated expression of these genes might contribute to disease progression through mechanisms such as excessive oxidative stress, impaired bioenergetics, and mitochondrial fragmentation [16, 17]. In contrast, genes such as NT5DC2, ATP5MC3, and GLYCTK exhibited protective effects, underscoring the importance of maintaining mitochondrial integrity and energy homeostasis [18]. These results align with previous studies highlighting the central role of mitochondrial health in diabetic complications.

Our single‐cell analysis provided cell‐type‐specific insights into mitochondrial dysfunction in the renal cortex of DKD patients. Notably, ATP5MC3 and NT5DC2 were downregulated in PODOs, while GLYCTK was downregulated in PCTs and LOH. PODOs are particularly susceptible to mitochondrial dysfunction due to their high energy demands, and disruptions in ATP5MC3 may lead to cytoskeletal abnormalities, foot process effacement, and eventual glomerular barrier loss [19, 20]. GLYCTK (glycerate kinase) plays a pivotal role in glyoxylate and dicarboxylate metabolism. While its specific functions in pPODOs are less well understood, GLYCTK deficiency may lead to the accumulation of metabolic byproducts, exacerbating oxidative stress and disrupting mitochondrial function. Oxidative stress is a key driver of PODO injury in DKD, contributing to cell apoptosis and detachment [21, 22]. NT5DC2 (5^′^‐nucleotidase domain‐containing protein 2) is implicated in nucleotide metabolism and energy regulation. Emerging evidence suggests that NT5DC2 also modulates immune responses and cellular proliferation. In PODOs, NT5DC2 downregulation may impair nucleotide homeostasis, increasing susceptibility to injury from inflammatory and metabolic stressors commonly observed in DKD [18, 23, 24].

The mediation MR analysis revealed that immune cells partially mediate the relationship between mitochondrial dysfunction and DKD. Specifically, HLA‐DR + pDCs, DCs, and CD33‐ HLA‐DR + immune cells were implicated in this pathway. DCs, as key antigen‐presenting cells, can amplify renal inflammation by recruiting T cells and sustaining tubulointerstitial immune infiltrates [25]. The proinflammatory actions of DCs contribute to tissue damage in various types of acute kidney injury and chronic glomerulonephritis, as DCs recruit and activate effector T cells, which release toxic mediators and maintain tubulointerstitial immune infiltrates [26, 27]. Our findings highlight the role of DCs and the interplay between mitochondrial dysfunction and immune cell activation in DKD. The increased presence of LEUKs in the renal cortex of DKD patients, as observed in our single‐cell analysis, further supports the involvement of immune‐mediated mechanisms in DKD. We also found HLA‐DR on CD33‐ HLA‐DR + was linked to DKD risk; however, few studies have addressed this association, which requires further exploration.

These findings indicate that mitochondrial dysfunction in PODOs, PCTs, and LOH cells may act as a trigger for immune cell infiltration in DKD. Although our MR results specifically implicated DCs as causal mediators, it remains unclear whether mitochondrial dysfunction within immune cells themselves also contributes to their infiltration and activation. As mitochondrial metabolism is essential for immune cell differentiation and effector function [28], further mechanistic studies are warranted to explore this possibility.

Increased infiltration of immune cells, including helper T cells, B cells, DCs, and plasma cells, was observed in the renal cortex of DKD patients. Although the cell–cell communication analysis identified PODOs, PCT, DCT, and endothelial cells as major hubs, LEUK‐related ligand–receptor signaling appeared relatively weak. This indicates that the contribution of immune cells to DKD pathogenesis may not primarily depend on direct ligand–receptor interactions with parenchymal cells but rather on enhanced infiltration and activation within the renal cortex [8, 26]. Importantly, this pattern is consistent with our observation that mitochondrial dysfunction in PODOs and tubular cells may generate metabolic stress that promotes immune recruitment and inflammatory activation [9, 29]. Thus, the mitochondria‐immune axis likely drives DKD progression predominantly through immune infiltration and systemic inflammatory signaling, rather than through stable local ligand–receptor crosstalk.

This proinflammatory environment was further supported by transcriptomic analysis of PBMCs, which identified upregulation of proinflammatory genes such as CXCL2, CXCL3 [30]. Elevated levels of DEFA3 indicate a strengthened antimicrobial defense, potentially as a response to increased infection or stress in DKD patients [31]. Increased expression of SMAD7 suggests altered TGF‐beta signaling, which might impact fibrosis and cellular responses in DKD [32]. These genes are associated with chemotaxis, fibrosis, and immune activation, all of which contribute to DKD progression. Targeting these inflammatory pathways could offer new avenues for therapeutic intervention.

Overall, our findings provide valuable insights into the interplay between mitochondrial dysfunction, immune cell infiltration, and the progression of DKD. By identifying key mitochondrial genes (e.g., ATP5MC3, GLYCTK, NT5DC2) and immune traits (e.g., HLA‐DR + DCs) associated with DKD risk, this study highlights potential therapeutic targets for early intervention. For instance, pharmacological strategies aimed at restoring ATP5MC3 function or modulating DC activity could potentially mitigate renal damage and slow disease progression. Moreover, the downregulation of mitochondrial genes in specific renal cell types underscores the importance of cell‐type‐specific therapies, which may improve the precision of DKD management. Future research focusing on small molecule inhibitors or gene therapies targeting these pathways could pave the way for innovative treatments to reduce the burden of DKD.

The main strength of the present study is that we conducted an extensive MR analysis to explore the causal relationship between mitochondrial dysfunction and DKD. By including all mitochondrial‐related genes, we minimized selection bias present in previous studies and directly addressed mitochondrial dysfunction. Utilizing MR allowed us to minimize confounding factors and reverse causation, providing more reliable evidence for the causal relationships between mitochondrial dysfunction, immune cell infiltration, and DKD. Multiple sensitivity analyses, including MR‐Egger regression, leave‐one‐out analysis, and Cochran’s Q test, were performed to ensure the validity and reliability of our findings. Additionally, validation in independent cohorts enhanced the robustness and generalizability of the results. We employed mediation MR to investigate the interplay between mitochondrial dysfunction and immune cell infiltration in DKD, offering deeper insights into their interconnected roles. Besides, the study uniquely combined MR and scRNA‐seq analysis, enabling robust causal inference and high‐resolution cellular insights. This integrative approach provided a comprehensive understanding of the complex mechanisms underlying DKD.

This study has several limitations. First, although extensive GWAS and eQTL resources were used, no variants directly reflecting mitochondrial protein expression or mitochondrial genome regulation were available. Some associations did not remain significant after multiple testing correction, and residual pleiotropy cannot be fully excluded. In addition, to ensure a sufficient number of instruments, we adopted a suggestive significance threshold (*p*  < 1 × 10^−5^) and an LD clumping threshold of *R* ^2^ < 0.1, which may increase the possibility of incorporating moderately associated or partially correlated variants. Second, the kidney cortex and scRNA‐seq datasets were limited in sample size, temporal resolution, and LEUK representation, which may not fully capture the heterogeneity of DKD. Third, while we identified ATP5MC3, GLYCTK, and NT5DC2 as downregulated in PODOs and tubular cells, their specific roles in mitochondrial dysfunction and immune crosstalk remain undefined. Fourth, we did not perform bidirectional MR due to the lack of strong and independent instruments for the outcome trait, and therefore reverse causation between mitochondrial pathways and DKD‐related traits cannot be fully ruled out. Despite these limitations, our findings highlight important directions for future research. Expanding genomic resources to include mitochondrial genome‐specific QTLs and direct measures of mitochondrial protein expression will be essential. Larger and longitudinal single‐cell datasets could better capture dynamic changes in mitochondrial function and immune infiltration during DKD progression. Functional validation of candidate genes‐through perturbation studies in cellular and animal models will be critical to establish causality and delineate the underlying signaling pathways. Ultimately, integrating these approaches may translate our genetic and transcriptomic observations into novel therapeutic strategies for DKD.

## 5. Conclusion

Our study offers novel insights into the intricate relationship between renal mitochondrial dysfunction, immune cell infiltration, and DKD. By combining integrative MR and scRNA‐seq analysis, we identified key mitochondrial genes and immune cell types involved in the pathogenesis of DKD. These findings deepen our understanding of disease mechanisms and highlight potential new therapeutic targets.

## Ethics Statement

All data used in this study were obtained from public databases, and ethics approval and informed consent documents were with the original study authors.

## Disclosure

Preliminary results were presented as an abstract at the 61st European Association for the Study of Diabetes (EASD) Annual Meeting (Vienna, 2025) [33], and a preprint of this work has been published [34]. All authors read and approved the final manuscript.

## Conflicts of Interest

The authors declare no conflicts of interest.

## Author Contributions

Chao Zheng, Riqiu Chen, and Jun Wang designed this study. Tianyue Zhang, Junxia Wu, Jiazhi Zhang, Yepeng Hu, Yiming Zhao, Guangyun Mao, and Jingjing Jiao collected and analyzed the data. Tianyue Zhang wrote the initial draft of the manuscript. Chao Zheng, Riqiu Chen, and Jun Wang supervised the study, developed the concept, and edited the paper. All authors have contributed significantly. Tianyue Zhang and Junxia Wu have contributed equally to this work and share first authorship.

## Funding

This research was funded by grants from the Zhejiang Provincial Natural Science Foundation of China (Grant LQ24H050002) and the National Natural Science Foundation of China (Grant 82100925).

## Supporting Information

Additional supporting information can be found online in the Supporting Information section.

## Supporting information


**Supporting Information 1** Figure S1: Causal Effects of Mitochondrial Gene Expression on DKD in the Replication Dataset.

**Supporting Information 2** Figure S2: Causal Effects of Immune Cells on DKD in the Replication Dataset.

**Supporting Information 3** Figure S3: Cell Type Identification. (A) Expression of lineage‐specific markers for cell type identification. (B) Features and identity plot for cell type identification.

**Supporting Information 4** Figure S4: Changes in Renal Cortex and PBMC of DKD Patients. (A) Increased number of LEUK cells in DKD kidneys. (B) Top three upregulated and downregulated DEGs in each kidney cell type in DKD. (C) Expression of ATP5MC3, NT5DC2, and GLYCTK in kidney cell types (same as Figure 4A) in DKD. (D) Expression of ATP5MC3, NT5DC2, and GLYCTK in kidney cell types (same as Figure 4A) in control kidneys. (E) Cell–cell interaction network in DKD kidneys. (F) Cell–cell communication pathways in DKD kidneys. (G) Volcano plot showing DEGs in PBMCs of DKD patients. (H) Functional enrichment analysis of DEGs in PBMCs of DKD patients.

## Data Availability

All data generated or analyzed during this study were included in this published article and its additional files.
