# Supplementary figures and images for: Mitochondrial Dysfunction and Immune Cell Infiltration in Diabetic Kidney Disease: A Mendelian Randomization and Multiomics Study
Source: Mediators Inflamm. 2025 Dec 25;2025:5592084. doi: 10.1155/mi/5592084 (PMC12767380; doi:10.1155/mi/5592084)

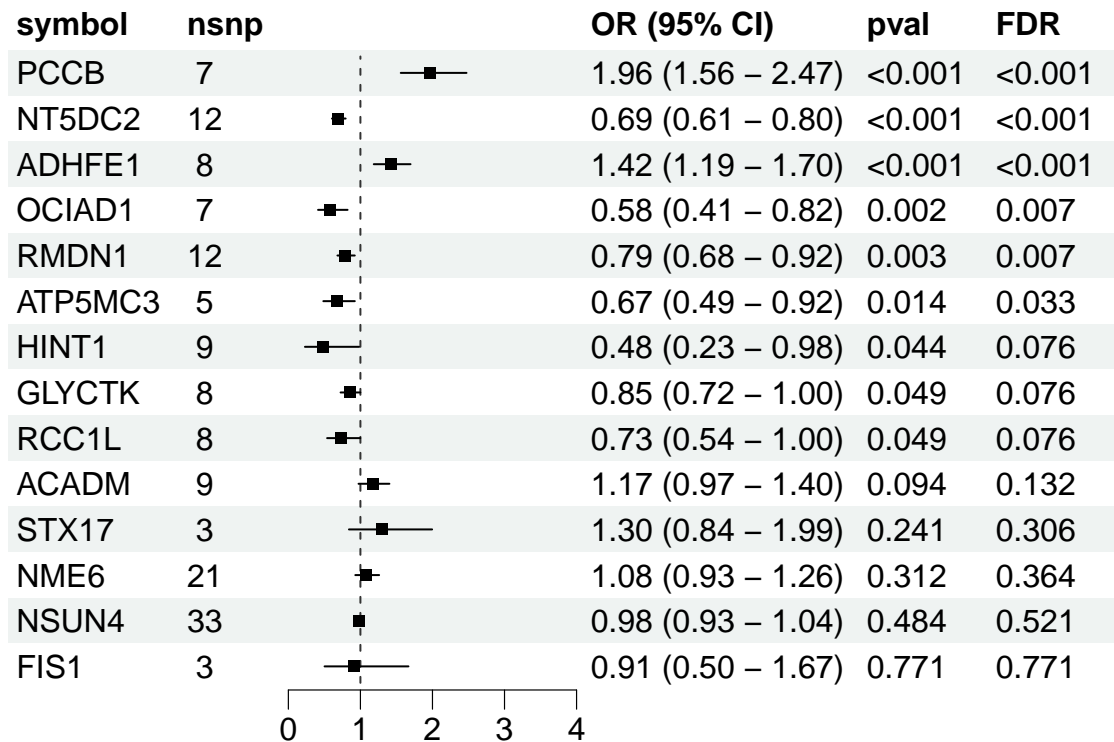

Supplement: Supplementary file 1 — Supporting Information 1 Figure S1: Causal Effects of Mitochondrial Gene Expression on DKD in the Replication Dataset. [file MI-2025-5592084-s001.pdf]

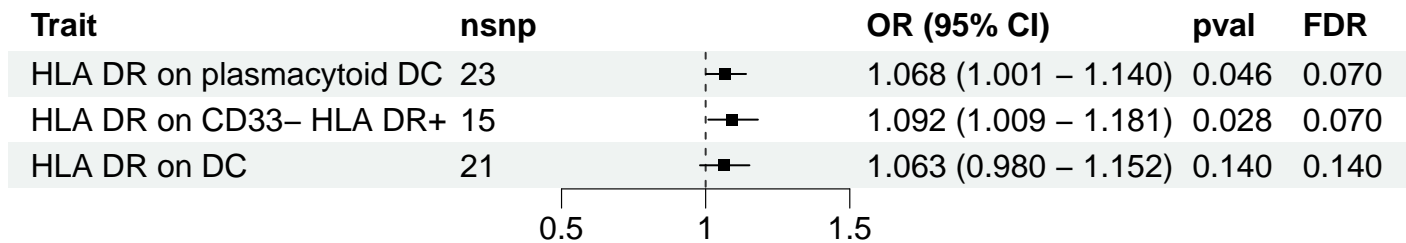

Supplement: Supplementary file 2 — Supporting Information 2 Figure S2: Causal Effects of Immune Cells on DKD in the Replication Dataset. [file MI-2025-5592084-s003.pdf]

A

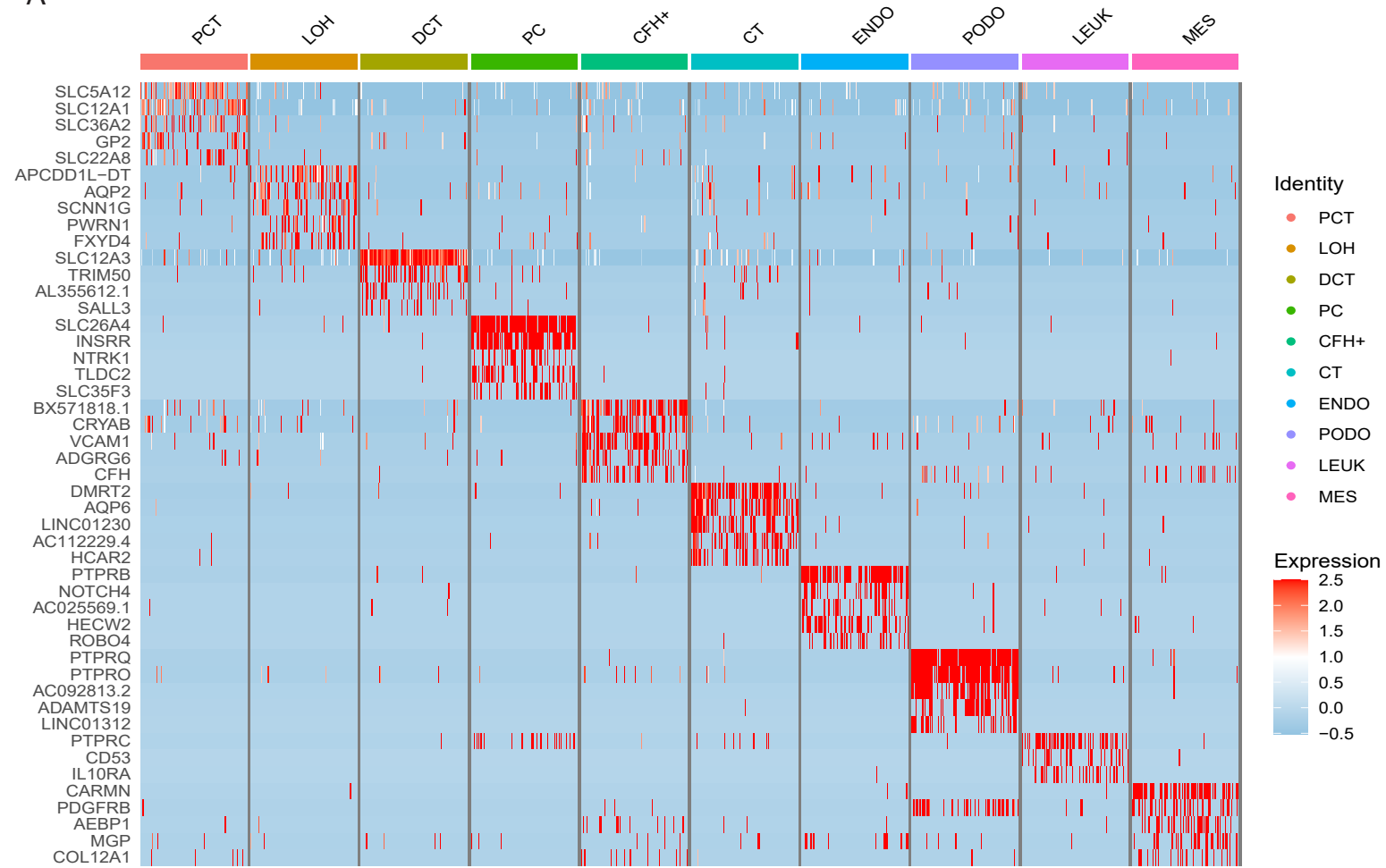

B

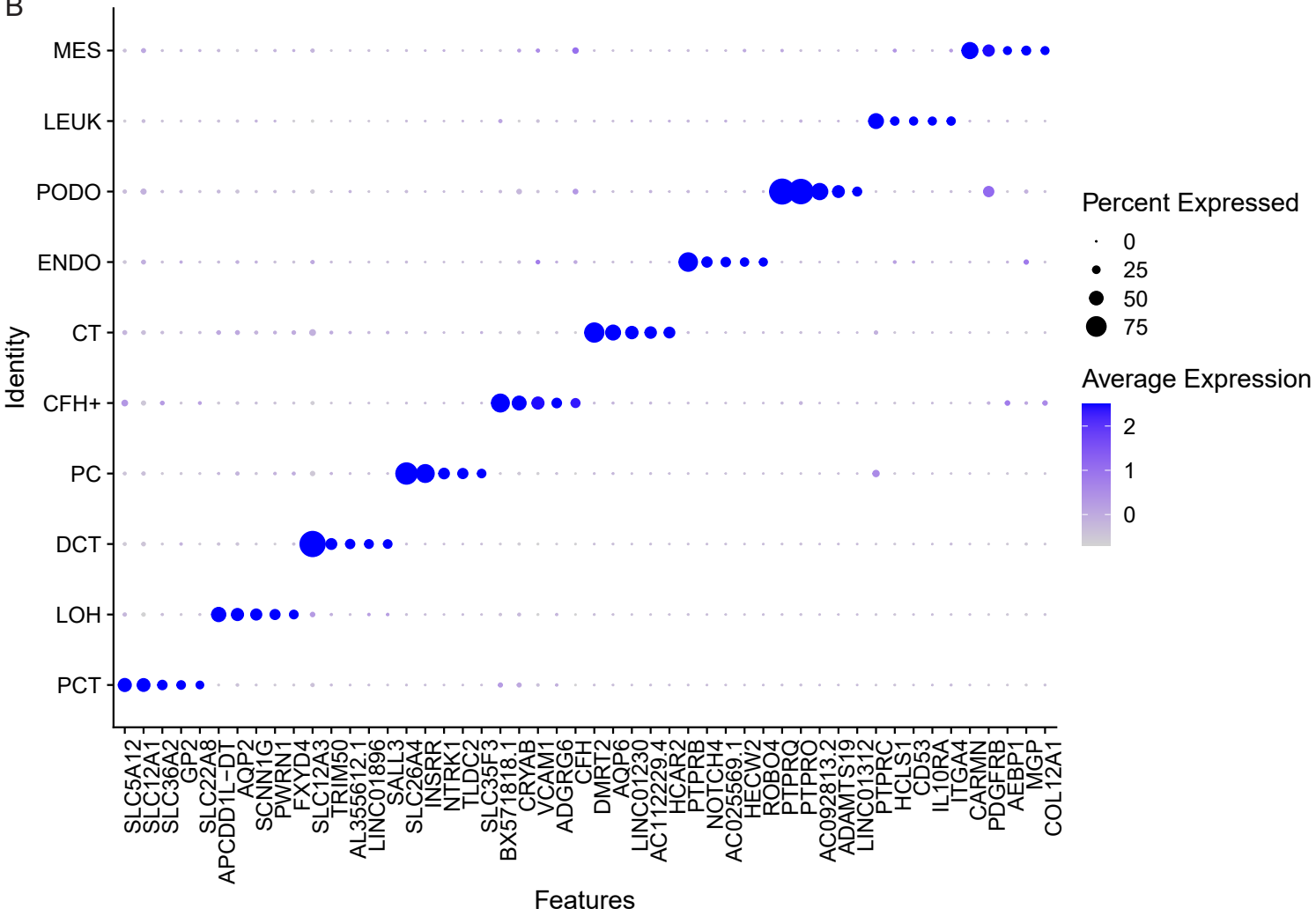

Supplement: Supplementary file 3 — Supporting Information 3 Figure S3: Cell Type Identification. (A) Expression of lineage‐specific markers for cell type identification. (B) Features and identity plot for cell type identification. [file MI-2025-5592084-s002.pdf]

A

Balloon Plot for x by y.  
Area is proportional to Freq.

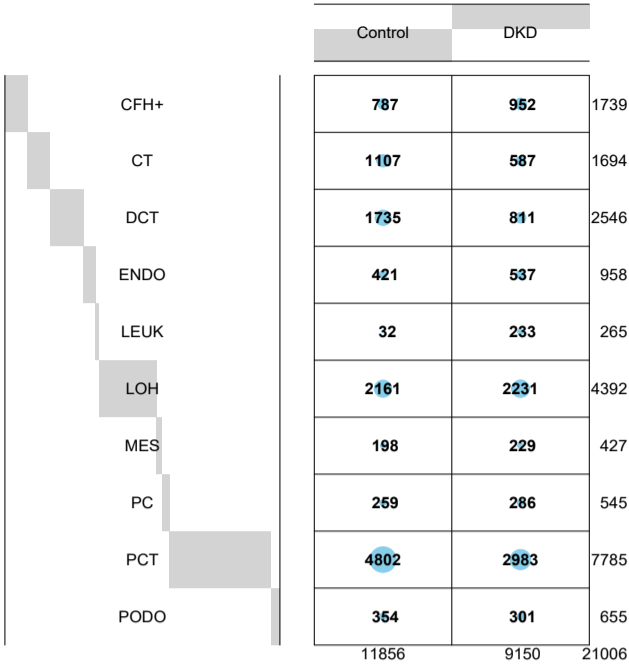

B

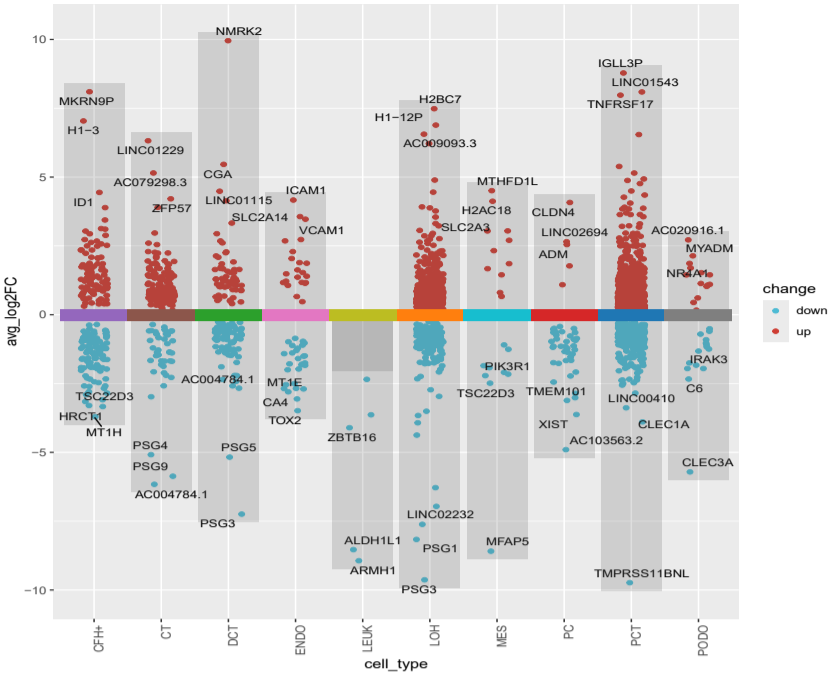

C

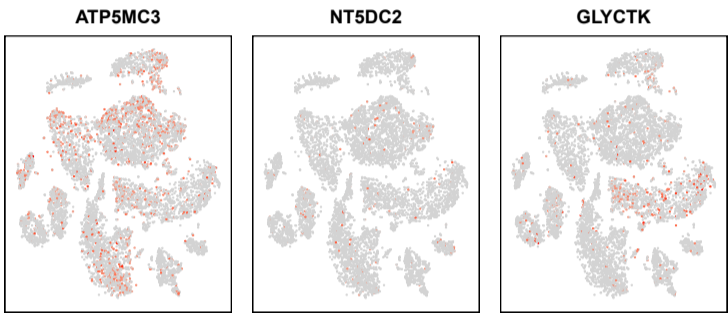

D

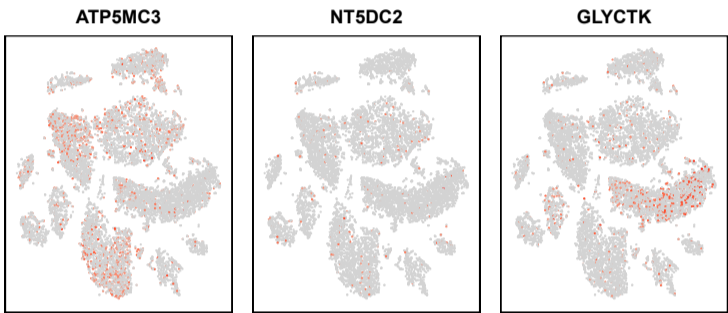

E

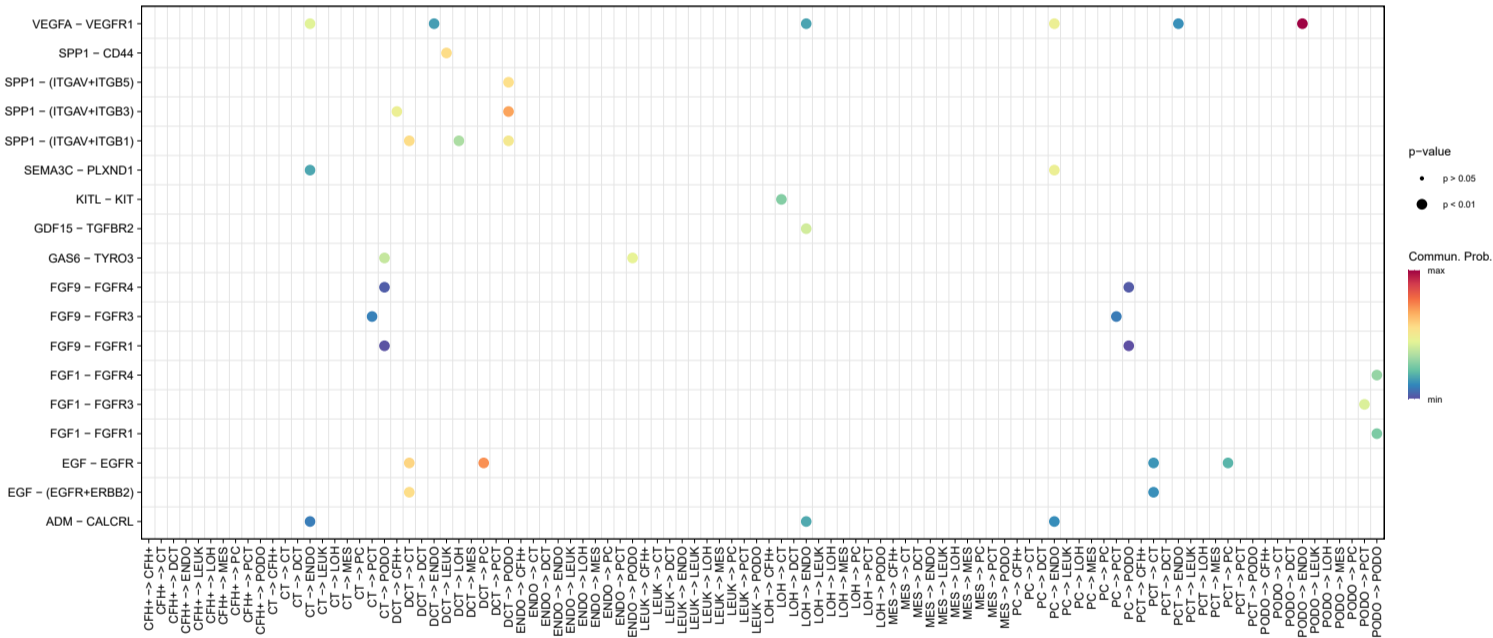

F

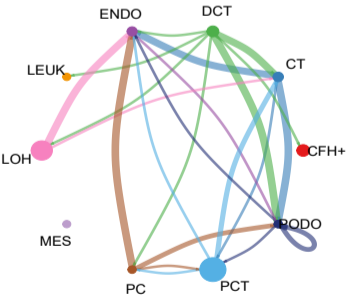

H

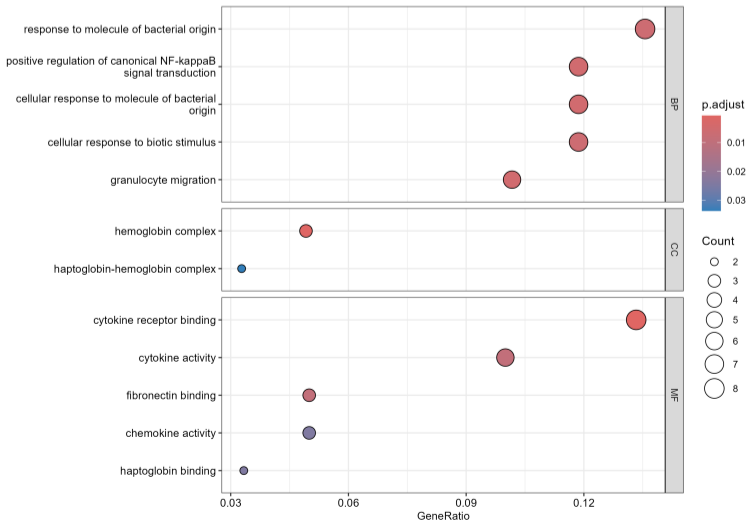

G

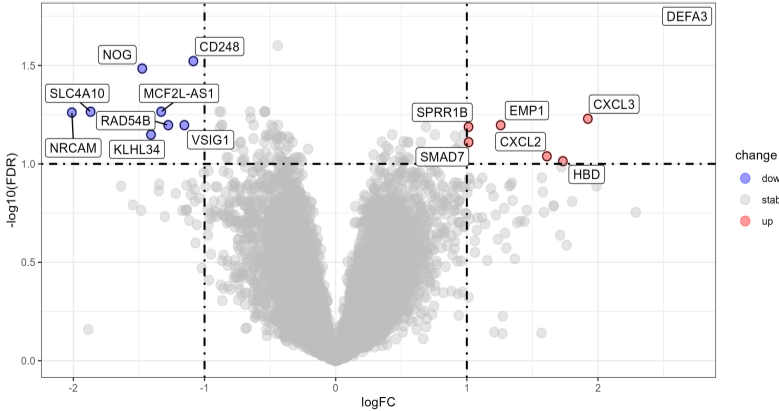

Supplement: Supplementary file 4 — Supporting Information 4 Figure S4: Changes in Renal Cortex and PBMC of DKD Patients. (A) Increased number of LEUK cells in DKD kidneys. (B) Top three upregulated and downregulated DEGs in each kidney cell type in DKD. (C) Expression of ATP5MC3, NT5DC2, and GLYCTK in kidney cell types (same as Figure 4A) in DKD. (D) Expression of ATP5MC3, NT5DC2, and GLYCTK in kidney cell types (same as Figure 4A) in control kidneys. (E) Cell–cell interaction network in DKD kidneys. (F) Cell–cell communication pathways in DKD kidneys. (G) Volcano plot showing DEGs in PBMCs of DKD patients. (H) Functional enrichment analysis of DEGs in PBMCs of DKD patients. [file MI-2025-5592084-s004.pdf]
